# Statistical parametric mapping of biomechanical one-dimensional data with Bayesian inference

**DOI:** 10.1080/23335432.2019.1597643

**Published:** 2019-04-04

**Authors:** Ben Serrien, Maggy Goossens, Jean-Pierre Baeyens

**Affiliations:** a Faculty of Physical Education and Physiotherapy, Vrije Universiteit Brussel, Brussels, Belgium; b Faculty of Applied Engineering, Universiteit Antwerpen, Antwerp, Belgium; c Thim Van Der Laan University College Physiotherapy, Landquart, Switzerland

**Keywords:** Bayesian inference, Bayes Factor, posterior probability, Statistical Parametric Mapping, time series, false discovery rate, Q-value

## Abstract

Recent developments in Statistical Parametric Mapping (SPM) for continuum data (e.g. kinematic time series) have been adopted by the biomechanics research community with great interest. The Python/MATLAB package spm1d developed by T. Pataky has introduced SPM into the biomechanical literature, adapted originally from neuroimaging. The package already allows many of the statistical analyses common in biomechanics from a frequentist perspective. In this paper, we propose an application of Bayesian analogs of SPM based on Bayes factors and posterior probability with default priors using the BayesFactor package in R. Results are provided for two typical designs (two-sample and paired sample *t*-tests) and compared to classical SPM results, but more complex standard designs are possible in both classical and Bayesian frameworks. The advantages of Bayesian analyses in general and specifically for SPM are discussed. Scripts of the analyses are available as supplementary materials.

## Introduction: Statistical Parametric Mapping

Statistical Parametric Mapping (SPM) was originally developed for statistical inference on neuroimaging data where dependent variables are sampled on a large number of spatially correlated voxels (volume elements) (Friston 2007)⁠. The same methodology applies, however, to all spatiotemporally registered and smooth data, and subsequent work from the research group of Todd Pataky has introduced SPM in the biomechanics and human movement science community for analysis of pedobarographic images (Pataky and Goulermas 2008)⁠, finite-element simulations (Pataky 2010)⁠ and uni/multivariate time series data (kinematics, kinetics, sEMG, etc.)(Pataky 2012; Pataky et al. 2013; Robinson et al. 2015)⁠. Recent developments include power analysis and sample size calculations (Pataky 2017) and SPM for cortical bone mapping (Li et al. 2009; Poole et al. 2017; Yu et al. 2017) and pedobarographic videos (Booth et al. 2018)⁠. The nomenclature of SPM uses ‘*nDmD’* to describe the dimensionality of the dataset, where the parameter *n* describes the dimension of the field in which the dependent variable(s) is(are) sampled and the parameter *m* describes the number of dependent variables (Pataky et al. 2016)⁠. In the present paper, our focus lies on univariate time series, i.e. *1D1D* data where one dependent variable is sampled continuously over time (a one-dimensional field), but the same principles apply to all *nDmD* data.

SPM offers a couple of strong advantages for biomechanists and movement scientists. The primary advantage is that no abstraction of the originally sampled time series needs to be performed in order to statistically analyze the data. The full *1D* field can be examined in a non-directed hypothesis test without any ad-hoc assumptions regarding the spatiotemporal foci of interest. Since kinematic or kinetic time series can be complex, it can be difficult to objectively specify an a-priori method for analysis and many studies, therefore, adopt an ad-hoc approach: visualize the *1D* time series and extract a summary *0D* scalar (extremum, central tendency, …) which was not specified a priori to test statistically (Pataky et al. 2015)⁠. Accompanying these full-field non-directed hypotheses tests is the ability to visualize the statistical results in the same field as where the data were sampled. For time series data, the statistical result is hence also a time series (e.g. a time series of *t*-values) and allows for better interpretation of data.

SPM uses Random Field Theory (RFT) (Adler and Taylor 2007)⁠ to perform topological inference instead of performing separate inferential tests at each time point which would cause an inflation of Type I error. RFT leverages smoothness (local correlation between adjacent time points) to mitigate the multiple testing problem, thereby offering accurate sampling-rate independent control of Type I errors when testing correlated field data. Because biological processes are typically smooth and biomechanical data acquisition samples above the Nyquist criterion, neighboring time samples are not independent and this should be taken into account (Pataky 2010)⁠. Rather than computing a *p*-value at each time sample, a *p*-value is calculated for clusters of statistics (e.g. *t*) that cross a critical threshold (t*). The logic of RFT is that the height and width of supra-threshold clusters produced by smooth random fields are inversely proportional to the probability of their occurrence, making a large supra-threshold cluster the topological equivalent of a large *t*-value for *0D* data (Pataky 2010; Appendix A)⁠. The definition of the SPM *p*-values can be stated as: ‘*the probability that smooth, random continua would produce a supra-threshold cluster as broad as the observed cluster*’ (spm1d.org, © T. Pataky). Critical thresholds are usually calculated with the Type I error α = 0.05. Hence, when the observed *t-*statistic time series crosses the threshold, this cluster has a *p* < 0.05, allowing the researcher to reject the null hypothesis *H_0_* of no difference between the two time series.

These SPM *p*-values – as in *0D* statistics – refer to the probability of the data given that *H_0_* is true, *P*(data | *H_0_*), without recourse to any alternative hypothesis *H_1_*, which is the classical frequentist approach to inference. In the current implementation of the open-source package *spm1d* (Python and MATLAB versions, spm1d.org, © T. Pataky), there is only the possibility to perform frequentist inference and in this paper, we want to propose a stepping stone towards a Bayesian alternative. In the following sections, we will first briefly introduce the differences between Bayesian and frequentist inference and delineate why the Bayesian alternative can offer additional insights from the data.

## Bayesian inference

Classical inference answers the inverse question of what researchers usually aim to answer. Above we gave the definition of a frequentist *p* which is not the same as what we want to know, namely the probability that *H_0_* or *H_1_* are good descriptions of the data: *P*(data | *H_0_*) *≠ P*(*H_0_* | data) (Cohen 1994)⁠. In fact, both probabilities are related to each other through Bayes’ theorem:

$$P(H_{i}|data)=\frac{P\left(data|H_{i}\right)P\left(H_{i}\right)}{\sum P\left(data|H_{j}\right)P\left(H_{j}\right)}$$

where the sum in the denominator (or integral in the limiting case) is taken over the set of all relevant hypotheses *j* (including *i*). Additionally, frequentist inference is asymmetric in the sense that: (1) it is only possible to state evidence against *H_0_* and not evidence in favor of *H_0_* or in favor of any alternative *H_1_* and (2) because it does not consider any alternative hypotheses, the evidence against the null is always overstated (Rouder et al. 2009; Morey and Rouder 2011; Wagenmakers et al. 2018)⁠. When a researcher wishes to demonstrate the invariance of some variable’s time series during a movement (invariance with respect to a model prediction, experimental manipulation or group membership), classical inference only allows statements like ‘the data showed no evidence against *H_0_* during the movement’ which is not the same as the statement which was the aim of the study: ‘the data showed evidence in favor of *H_0_* and thus an invariance during the movement’. With Bayesian inference, the latter statements are possible. For instance, consider a study where the objective is to show that gait kinematic time series are left-right symmetric, Bayesian inference can quantify the evidence in favor of the *H_0_: μ*_left_(t) *= μ*_right_(t). Conversely, a classical approach would be to assume that symmetry exists, calculate a *p*-value under this assumption and fail to reject the null. But it makes no logical sense to assume something which you want to prove. Interested readers in further contrasts between classical and Bayesian inference are referred to recent tutorial papers on Bayesian statistics (Dienes and Mclatchie 2018; Etz and Vandekerckhove 2018; Kruschke and Liddell 2018a)⁠.

Within the Bayesian school of statistics, many related approaches exist, but in this paper, we will focus on an approach to Bayesian SPM based on Bayes Factors and Posterior Probability. A few other Bayesian alternatives are explained in the discussion. Bayes Factors (*BF*) result from the application of Bayes’ rule and can be linked to the odds of one hypothesis over another:

$$\underset{posteriorodds}{\underbrace{\frac{P(H_{1}|data)}{P(H_{0}|data)}}}=\underset{BF_{10}}{\underbrace{\frac{P(data|H_{1})}{P(data|H_{0})}}}.\underset{priorodds}{\underbrace{\frac{P(H_{1})}{P(H_{0})}}},$$

where *BF_10_* is the Bayes Factor with the marginal likelihood of the data under the alternative *H_1_* in the numerator and the likelihood under *H_0_* in the denominator. The prior odds reflect the relative belief in both hypotheses before doing the experiment and is often set equal to 1 in order not to favor any hypothesis a priori, in which case *BF_10_* reflects the posterior odds of the alternative over the null. However, it is not necessary to set the prior odds to 1, researchers may simply communicate the *BF* which readers may multiply with any prior odds they hold on the two competing hypotheses to yield a posterior odds. The *BF* reflects the change in confidence on the two hypotheses after observing the data (Wagenmakers et al. 2018)⁠. A *BF_10_* of 1 indicates equal evidence for both hypotheses while 0 < *BF_10_* < 1 indicates evidence in favor of *H_0_* and *BF_10_* > 1 is evidence in favor of *H_1_*. For instance, with the prior odds set to 1, a *BF_10_* of 5 indicates that *P*(*H_1_* | data) = 5/6 and *P*(*H_0_* | data) = 1/6, i.e. the probability of the alternative is 5 times higher than that of the null.

To calculate the Bayes Factor, researchers need to specify likelihood functions and associated prior probabilities for both hypotheses. This allows for very flexible analyses and to incorporate any prior knowledge about the specific data from experience, pilot studies, meta-analyses and other sources (Wagenmakers et al. 2018)⁠. The resulting *BF* is naturally sensitive to this choice, and it should be argued why a particular choice is relevant and how robust the results are with respect to reasonable changes in the prior setting. The so-called objective Bayesian school has developed default priors for a variety of typical statistical tests for which the resulting *BF* has desirable theoretical properties. These properties include: (1) *scale invariance* which means the default *BF* is unaffected by multiplicative changes of the variables (i.e. independent of the measurement units); (2) *consistency*, which means that in the large sample limit the *BF* will approach zero or infinity when the effect size is 0 or not zero, respectively; and (3) *consistency in information*, which indicates that the *BF* will approach the correct limit as the statistic of interest (e.g. *t*) increases:$\lim_{t\to \infty }BF_{10}=\infty$, independent of sample size (Rouder et al. 2012)⁠. These default priors are general and broadly applicable and are reasonable in most circumstances (Rouder et al. 2012)⁠ and we will, therefore, choose them for our proposal of a Bayesian implementation of SPM.

The *BF* based on default priors is a convenient summary of the evidence but has one disadvantage for SPM, namely the control of the multiple testing problem across the *1D* field (which typically includes 101 time points; 0–100% of the movement). However, the *BF* can be converted to posterior probabilities which are better suited to implement a multiple testing control scheme. Posterior probabilities are also easier to interpret for researchers used to classical statistics as they live on the [0, 1] interval whereas a *BF* exist on the]0, ∞[interval. Given a prior odds of 1, the posterior probabilities (*PP*) can be calculated as:

$$P_{H0}=P\left(H_{0}|data\right)=\frac{1}{1+BF_{10}}\;and\;PP_{H1}=P\left(H_{1}|data\right)=\frac{1}{1+BF_{01}}$$

When *PP_H1_* at a certain time point is, e.g. 0.95, the posterior error probability of classifying this time point as evidence in favor of *H_1_* is 0.05 (*PEP*= 1 *– PP*). The false discovery rate (FDR) can be used as a unified multiple-testing framework for Bayesian and classical inference (De Villemereuil et al. 2014)⁠ and is therefore adopted here. A conservative control of the FDR can be made by thresholding the posterior probability SPM at, e.g. 0.95, keeping the FDR ≤ 0.05 (Friston and Penny 2003)⁠. A less conservative threshold, while still keeping the FDR at the same level is the use of the *q*-value which is defined as the cumulative mean of the posterior error probabilities (Storey 2003; Käll et al. 2008)⁠. A *q*-value of 0.05 for a certain time point implicates that for all possible thresholds, 5% is the minimal FDR threshold at which this time point will appear in a supra-threshold cluster (Käll et al. 2008)⁠. Especially for SPM, the use of the *q-*value is, we believe, better suited, because it indirectly takes the temporal correlation of adjacent time points into account. Although a *q*-value is calculated per time point for *1D* data, it is a property of the entire time series object. When a threshold of q* = 0.05 is used, the first time point to be included in a supra-threshold cluster has at least a posterior probability of 0.95; adjacent time points may fall below that while still keeping below q*, which is reasonable because adjacent time points are strongly correlated and hence may be categorized in the same cluster.

## Classical SPM versus Bayesian SPM

In this section, we will compare the classical SPM with our proposition of a Bayesian version of Statistical Parametric Mapping for *1D1D* data.

### Datasets

We will use example datasets that come included with the open-source spm1d-package (spm1d.org, © T. Pataky) and one additional dataset from our lab. We will use common statistical tests for demonstration purposes (two-sample *t-*test and paired-sample *t-*test), but more complex statistical models are available in the packages for classical and Bayesian analysis (n-way ANOVA, repeated measures, (multi-)linear regression, …). A description of the three data-sets is given in Table 1.

**Table 1.** Description of example datasets used for the three frequentist and Bayesian SPM tests. The first two datasets are part of the spm1d-package (© T. Pataky)

Statistical test	Example dataset
Two-sample *t-*test	SimulatedTwoLocalMax. Dataset of 2 x n = 6 simulated time series of 101 time samples each. The first set are smooth unit Gaussian random trajectories. The second set are also smooth unit Gaussian trajectories, but with bursts at t = 25 and t = 75.
Paired-sample *t-*test	PlantarArchAngle (Caravaggi et al., 2010). Dataset of 2 x n = 10 experimental time series of the plantar arch angle of the foot at 101 time samples each.
Paired sample *t*-test	GaitSymmetry. Single subject dataset (healthy male, 28 years, 83 kg, 178 cm) of left and right leg knee flexion angles during 99 gait cycles on a dual-belt tredmill at constant speed of 4.5 km/h, time normalized to 101 time samples. Gait kinematics were recorded with a 6-camera VICON system at 250 Hz. (unpublished data from our lab).

### Classical SPM

The *spm1d* package was used to perform two-tailed SPM{*t*} tests in Spyder (Python 3.6). The classical null hypotheses for the three examples are as follows:

- SimulatedTwoLocalMax:  Independent-sample *t-*test:*   H_0_: μ_1_*(t) *= μ_2_*(t)
- PlantarArchAngle:  Paired- sample *t-* tests:*  H_0_: μ_1_*(t) *= μ_2_*(t)
- GaitSymmetry:   Paired-sample *t-*tests:*  H_0_: μ_left_*(t) *= μ_right_*(t)

**Figure 1.**
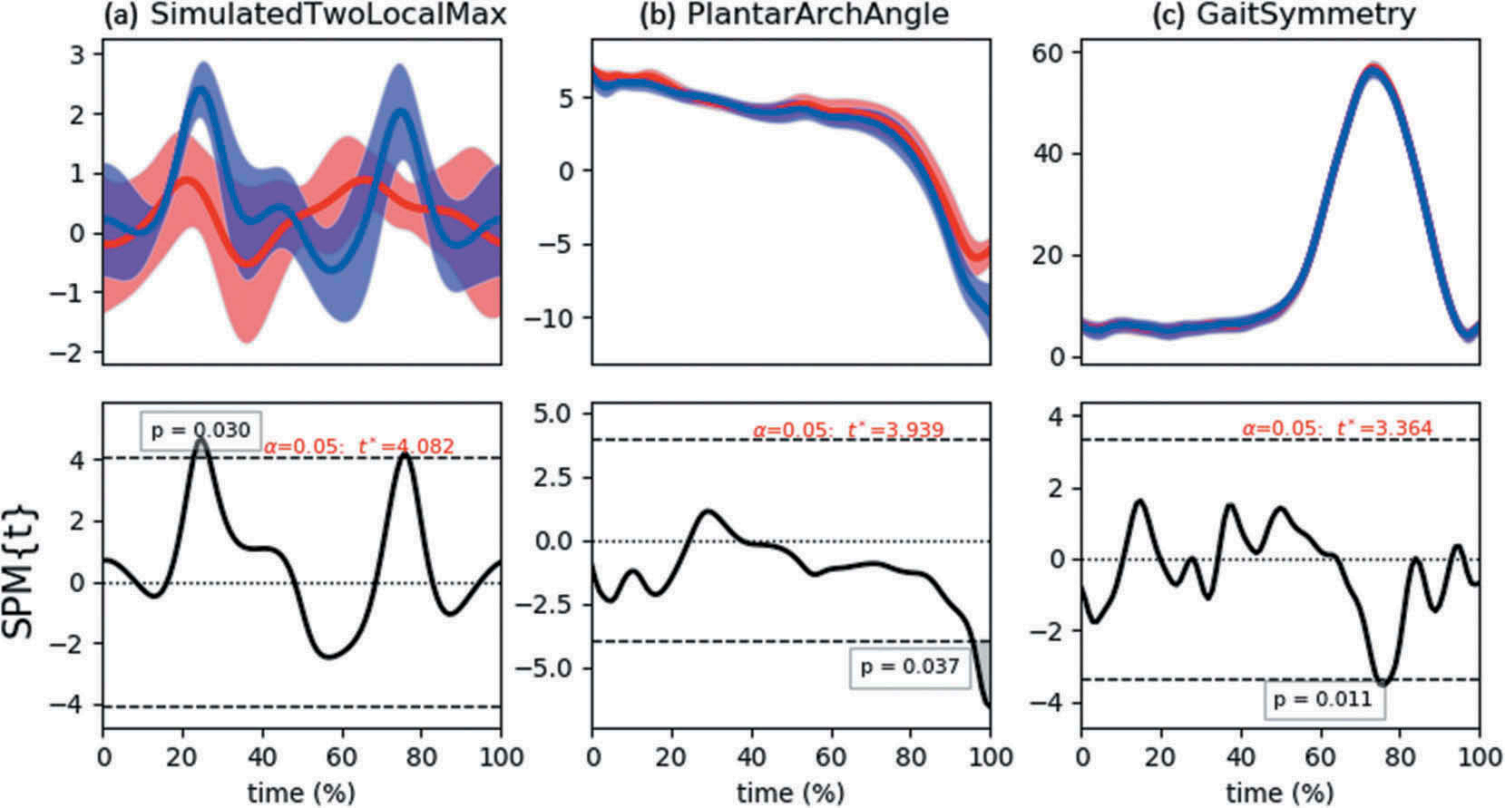
Classical SPM{*t*} results for the three datasets. Top row shows descriptive statistics for each dataset (Mean ± 1 SD error cloud). Bottom row shows the frequentist inferences. The horizontal dashed lines depict the critical t* based on *α* = 0.05 and RFT calculations of residual smoothness. Supra-threshold clusters result in *p* < 0.05. For the GaitSymmetry example, note that these are time series from a single subject, the SD-cloud thus represents within-subject variability instead of between-subject variability. The inference only pertains to this subject

The results of the analyses with *α*= 0.05 are shown in Figure 1, and the details of the supra-threshold clusters are depicted in Table 2.

**Table 2.** Classical SPM{t} results for the three datasets. Begin and end-points of supra-threshold cluster locations are given as a percentage of the total movement time

	Evidence against *H_0_ (p < 0.05)*	
	Cluster location	*p*-Value
SimulatedTwoLocalMax Independent-samples *t*-test	t = 24–27 t = 77	*p* = 0.030 *p* = 0.046
PlantarArchAngle Paired-samples *t*-test	t = 97–101	*p* = 0.037
GaitSymmetry Paired-samples *t*-test	t = 74–78	*p*= 0.011

In all three examples, *H_0_* may be rejected at a type I error rate of 5%. However, the supra-threshold clusters are quite small and specific to certain phases of the motion which is in contrast to the hypotheses stated above which apparently hold for all time points. In the PlantarArchAngle and especially for the GaitSymmetry example, it is contra-intuitive to completely reject the *H_0_* because the time series show a strong similarity. The GaitSymmetry dataset is a good example where a marginal effect can result in a statistically significant difference purely because a large sample size is obtained (but see results below on power). In the Bayesian case, this is not necessarily so and allows a more nuanced statement where the null can also be accepted in other time spans.

### Bayesian SPM: posterior probability maps

Similar to the classical approach, we will construct a Statistical Parametric Map of the posterior probability in the time domain where *P*(*H* | data) is calculated at each time sample, analogous to an SPM{*t*}. The calculations of the Bayes Factors and posterior probabilities are performed in RStudio with the R-package BayesFactor (Morey et al. 2018)⁠, scripts and datasets are available as supplementary materials.

The hypotheses and priors for the *t*-tests are parameterized in terms of the effect size *δ =*(*μ_1_ – μ_2_*)/*σ*, where the indices refer to the two groups or two conditions or left and right legs. Point null hypotheses are very unlikely to be true exactly and trivially small effects may exist that are not of (clinical, theoretical) interest. This does not mean that the null should be abandoned and it may still be preferred for parsimony’s sake as a first approximation of the truth (Cohen 1994; Morey and Rouder 2011)⁠. Morey and Rouder (2011) provide *BF* calculations where the null includes trivially small effects around *δ* = 0. We believe that this is especially appropriate for *1D* time series data (and increasingly so for higher dimensional fields) because point null models would already be unlikely due to technical data registration issues and natural movement variability. In the calculations below, we took [−0.2, 0.2] as an interval of trivially small effects for the difference in the time series data, this choice corresponds to the typical recommendation by (Cohen 1988)⁠ for small effects. The null and alternative hypotheses for the Bayes Factor calculations were as follows:

- paired and independent samples *t*-tests (point *H_0_):*

*H_0_ : δ*(t) = 0

*H_1_ : δ*(t) ~ Cauchy(*r*)

- paired and independent samples *t*-tests (interval *H_0_*):

*H_0_: δ*(t) ~ Cauchy(*r)* for *δ*(t) ∈ [−*c, c*]

*H_1_: δ*(t) ~ Cauchy(*r*) for *δ*(t) ∉ [−*c, c*]

**Figure 2.**
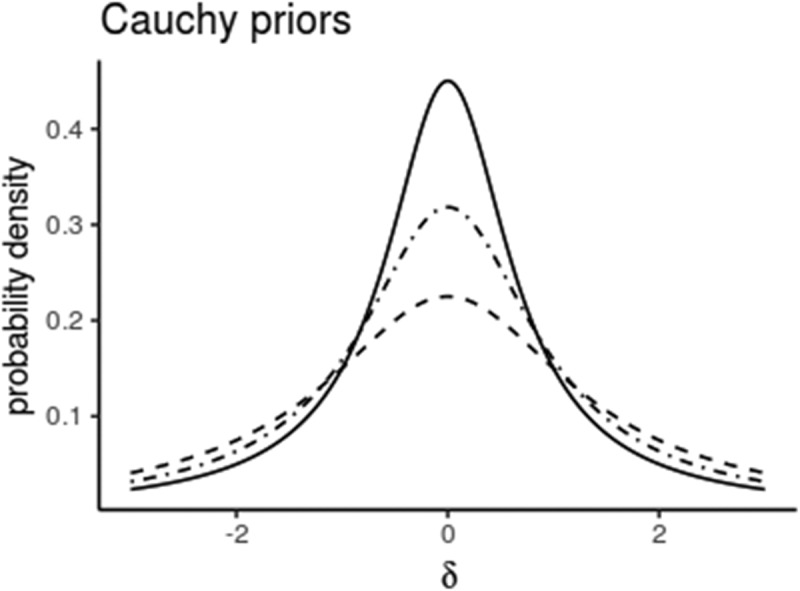
Cauchy priors for the effect size *δ* with different scales (solid line: *r* = √2/2 (medium), dot-dashed: *r* = 1 (wide) and dashed: *r* = √2 (ultra-wide)). Fifty percent of the probability mass lies between – *r* and + *r.*

Mathematical definitions of the default JZS-priors (Cauchy distributions), their justification and proofs for deriving the *BF*s can be found in (Rouder et al. 2009; Morey and Rouder 2011)⁠. These default priors still allow a flexible scaling of the width of the prior (*r*) and a determination of the null interval (*c*). The scale of the Cauchy prior should be set a priori and should reflect prior knowledge about the effect sizes that are relevant or expected for the variables of interest. The BayesFactor package offers three default options, that are shown in Figure 2. When the effect size is likely to be small to moderate, then the medium scale is a suitable choice, but when very large effects are expected, less probability should be placed in the center and more at the edges. For time series applications, a well-justified prior knowledge may even be reflected in phase-specific priors where the scale is a function of time, *r*(t). One-sided priors can also be selected for directed alternative hypotheses.

**Figure 3.**
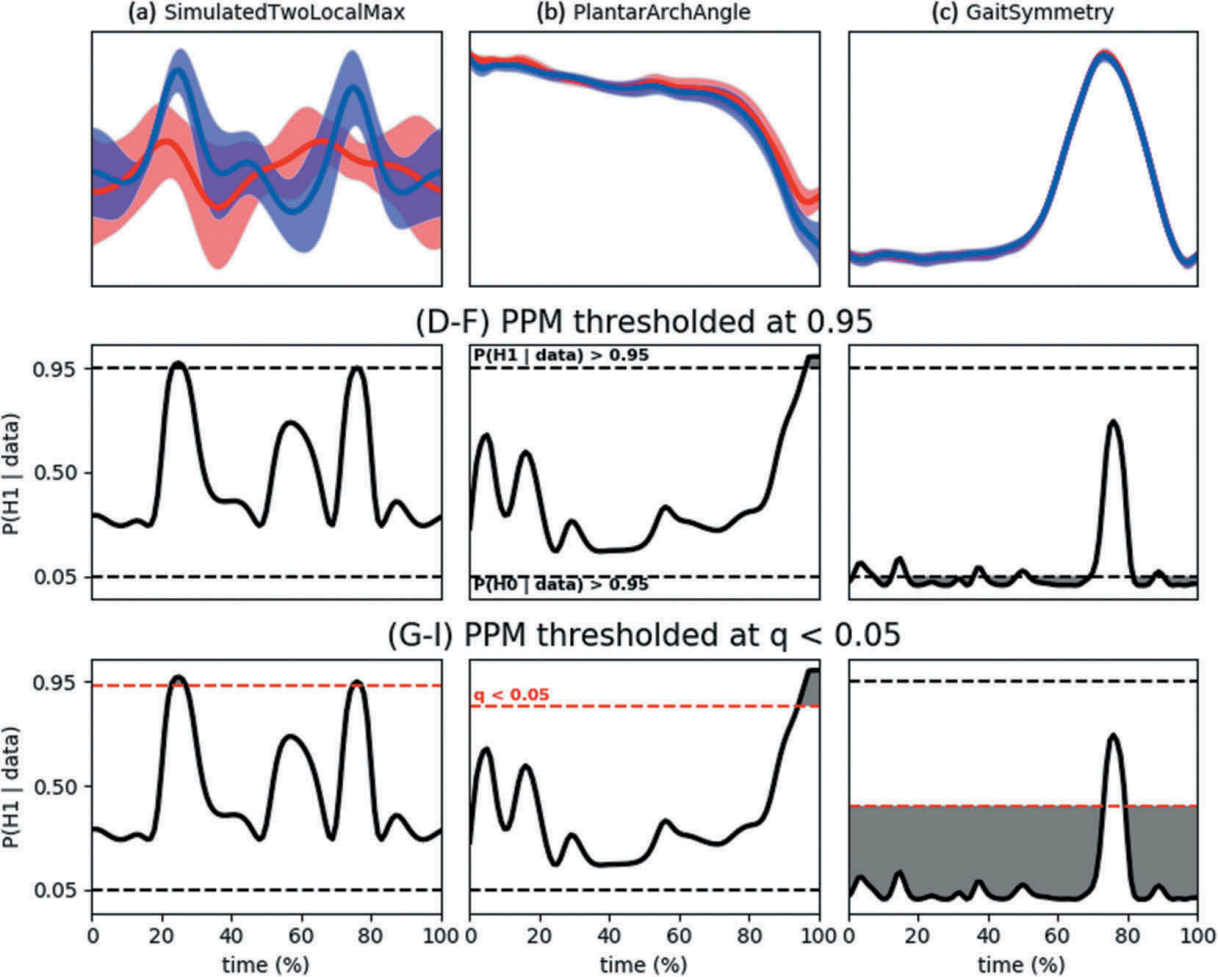
Panels (a), (b) and (c) give descriptive statistics for the three datasets (replicated from Figure 1). Panels (d), (e) and (f) give the posterior probability maps (PPM) for the alternative hypothesis: a time series of P(*H_1_* | data) (only shown for *r*= √2/2, see Table 3 for comparison to the other scales). The horizontal dashed lines at 0.05 and 0.95 depict the thresholds for which, respectively, P(*H_0_* | data) > 0.95 and P(*H_1_* | data) > 0.95 [P(*H_0_* | data) + P(*H_1_* | data) = 1]. Panels (g), (h) and (i) show the same PPM but thresholded using the FDR scheme. The red horizontal dashed line indicates the largest posterior error probability for which *q* < 0.05. It can be seen that no new clusters are created because the minimal posterior probability for either hypothesis must still be 0.95 in order to keep the *q* below 0.05. Because the cumulative mean is taken, the clusters broaden or in case of the GaitSymmetry dataset, they merge

In Figure 3 and Table 3, we show the results of the Bayesian SPM (posterior probability maps). Because we believe the interval *H_0_* to be the most relevant, we report only these results, but the reader may examine the point *H_0_* in the R-scripts (supplementary materials).

**Table 3.** Overview of supra-threshold clusters for the Bayesian SPM tests (interval *H_0_* only). The less conservative q* = 0.05 threshold always yields broader clusters than the *P*(*H* | data)* = 0.95 threshold. For the SimulatedTwoLocalMax and PlantarArchAngle datasets, the difference between both thresholds is small. For the GaitSymmetry example, the difference is larger and results in 4–7 separate clusters or 2 broad clusters (for the ultra-wide setting, it is nearly 1 cluster over the entire time span). The GaitSymmetry example also shows sensitivity to the scale of the prior, whereas this sensitivity was negligible in the other two datasets

	Evidence in favor of *H_0_*		Evidence in favor of *H_1_*	
	P(*H_0_* | data) ≥ 0.95	*q*(*H_0_*) ≤ 0.05	*P*(*H_1_* | data) ≥ 0.95	*q*(*H_1_*) ≤ 0.05
SimulatedTwoLocalMax (independent-samples *t*-test)				
*r*= medium	/	/	t = 25–27 t = 77	t = 24–28 t = 76–78
*r*= wide	/	/	t = 24–27 t = 77	t = 24–28 t = 75–78
*r*= ultra-wide	/	/	t = 24–27 t = 76–78	t = 24–28 t = 75–79
PlantarArchAngle (paired-samples *t*-test)				
*r*= medium	/	/	t = 98–101	t = 95–101
*r*= wide	/	/	t = 98–101	t = 95–101
*r*= ultra-wide	/	/	t = 98–101	t = 96–101
GaitSymmetry (paired-samples *t*-test)				
*r*= medium	t = 1–2 t = 9–13 t = 18–37 t = 41–49 t = 53–70 t = 83–88 t = 92–101	t = 1–74 t = 80–101	/	/
*r*= wide	t = 1–2 t = 8–14 t = 18–37 t = 40–50 t = 52–71 t = 83–101	t = 1–75 t = 79–101	/	/
*r*= ultra-wide	t = 1–3 t = 6–14 t = 17–71 t = 83–101	t = 1–76 t = 78–101	/	/

The results of the Bayesian SPM were partially in line with the classical SPM approach. For the independent-sample *t*-test, the classical SPM showed evidence against the null at t = 24–27 and at t = 77. The Bayesian SPM confirmed the existence of both clusters. For the conservative threshold, the results were nearly identical to the classical SPM, while the supra-threshold clusters were a little wider for the q* based threshold. Note that the posterior probability is below ½ most of the time, which makes sense given the model that created these simulated time series (see Table 1). However, the small sample size was ineffective for claiming strong enough evidence for accepting the null. For both types of threshold, the result was only weakly dependent on the width of the Cauchy scale.

For the PlantarArchAngle *t*-test, the classical SPM showed a significantly different plantar arch angle between t = 97–101, while the Bayesian supra-threshold cluster was a little wider (t = 95/96–101). Similar to the previous example, the sample size was too small to claim strong evidence in favor of the null, although the posterior probability was below ½ most of the time. From a Bayes Factor perspective, the null was more than 4 times more likely than the alternative at large phases of the gait cycle (*BF_10_* < ¼), but this was not enough to reach the posterior probability thresholds of 0.95. Also, for this example, the sensitivity to the prior scale was very small.

Arguably the biggest difference between both inferential perspectives lies in the GaitSymmetry dataset. From a classical perspective, the (point) *H_0_* could be rejected between t = 74–78 (*p*= 0. 011), whereas the Bayesian analysis only yields a maximum of 72% probability for the alternative during this time span, which is not convincing evidence for asymmetry. The Bayesian perspective shows, however, that throughout most of the time *P*(*H_0_* | data) *≥* 0.95. Using the q* threshold, we would say that except for a small amount of time (between 1% and 6% of the gait cycle), this subject is left-right symmetric in the knee joint motion. For this dataset, the results are more strongly dependent on the scale factor. Because the wide and ultra-wide settings place a more prior probability on large effect sizes, they are less likely alternatives and thus get penalized in favor of the null hypothesis which results in broader clusters.

**Figure 4.**
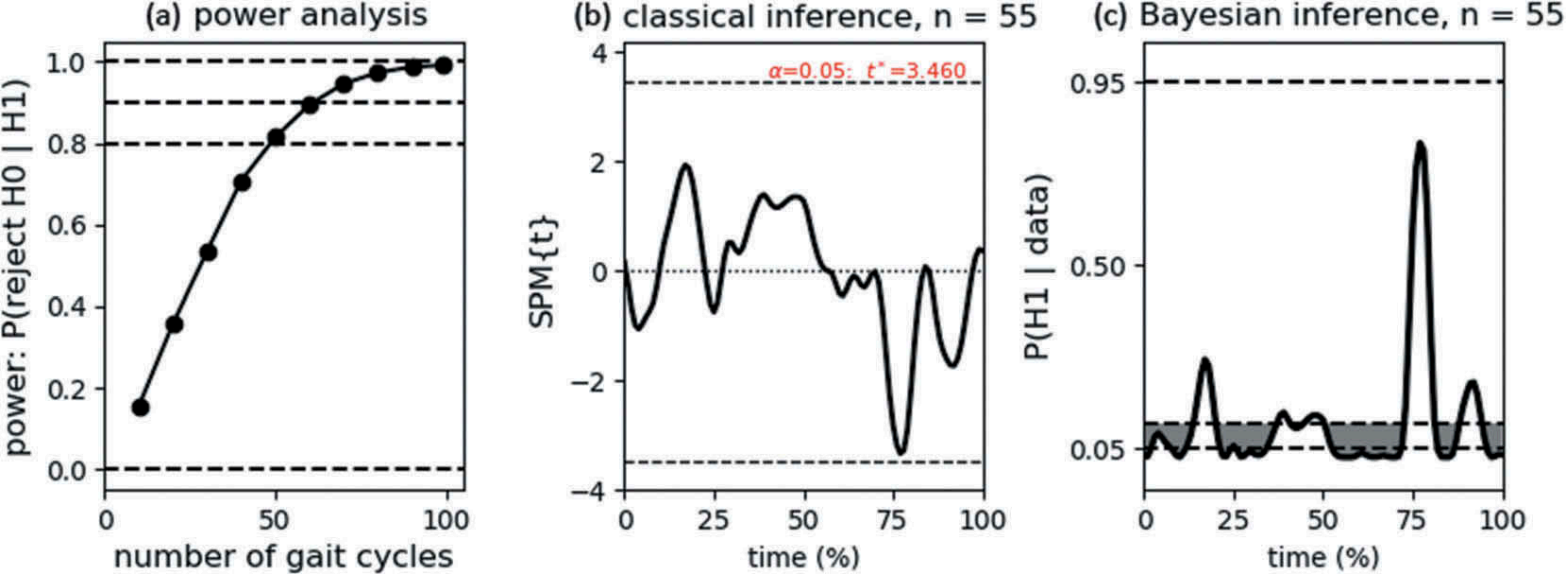
(a) Classical power (omnibus) analysis for calculating the number of trials necessary to reject *H_0_* given alpha = 0.05 and a minimal 2° difference between the left and right leg (*H_1_*). Horizontal lines show the typical power criterions of 0.80 and 0.90. Panels (b) and (c) give the classical and Bayesian SPMs using the first n = 55 gait cycles (for which classical power >0.80). For the Bayesian SPM, the maximal posterior error probability for which *q* < 0.05 was 0.113

Note, however, that in the classical SPM, the significant result is most likely caused by an overpowered dataset. This is a relatively common problem in single-subject designs where additional trials are easy to sample. The maximal significant mean difference was only 0.54° which is not clinically relevant to consider asymmetric. The present paper is only demonstrative, but if this were a proper experimental study, an a-priori power analysis may have helped to determine the number of gait cycles. Given a minimal difference of 2° to consider asymmetric, we performed a power analysis with the *power1d* package (Pataky 2017)⁠. The python script in the supplementary material explains the construction of the null and alternative models used for the simulations. The results showed that for a power of 0.80, minimal 50 gait cycles should be sampled (Figure 4(a)). Other simulations fluctuated a little around n = 50, so we performed the paired-samples *t*-tests again with the first 55 gait cycles, the results are shown in Figure 4(b) (classical) and 4C (Bayesian). The classical result no longer rejects *H_0_* while the Bayesian result still provides evidence for *H_0_* throughout most of the gait cycle. Technically these conclusions are not the same, but from an applied perspective, both conclusions would be in favor of symmetry.

## Discussion and future work

In this paper, we have proposed a stepping stone towards a Bayesian alternative to Statistical Parametric Mapping of *1D1D* data. We have shown results of posterior probability maps in two common statistical tests (two- and paired-sample *t*-tests) and compared the results to the classical SPM{*t*}. Both similarities and discrepancies are found between both inferential methods. Bayesian methodology in general (not only for *1D* data and SPM) has a stronger face validity and is not asymmetric like classical inference and takes an explicit alternative hypothesis into account which allows to calculate evidence in favor of a null hypothesis. We used an example of gait analysis to show how a Bayesian approach can statistically demonstrate that knee joint angles are left-right symmetric throughout nearly the entire gait cycle (single subject design). While an appropriately powered classical inference found no evidence of a significant left-right difference, this absence of evidence is not the same as a quantification of the evidence in favor of symmetry like in the Bayesian approach. This ability may also important in cases like designing neuromuscular models or testing theories where the time series of the prediction is compared to observed time series, *H_0_: μ_model_*(t) *= μ_empirical_*(t). Also in applied research with biomechanical time series, it may be relevant to examine invariance with respect to certain interventions. When some clinical or sports training intervention is performed in order to change the motion pattern, a frequentist approach could reject (successful intervention) or fail to reject the null (unsuccessful experiment), while a Bayesian approach could also provide evidence that the intervention itself is unsuccessful which is different from an unsuccessful experiment.

Classical sample size calculations for *0D*-data requires a definition of the minimal effect size that should be detectable with sufficient power and a given alpha level. For *1D*-data, null and alternative models should be constructed that represent the expected behavior of the time series under the null and alternative models. For well-known signals with simple behavior, this is relatively easy with the power1d package (Pataky 2017) but quickly becomes much more difficult for complex, unknown signals. Also, the expectation of the signal variability (within- and between subjects) and how and when the two groups would be different may be difficult to anticipate (effect cluster shape, height, width, location). When the observed effects in the final study are markedly different from the anticipated effects, the study may result in serious over/underpowered conclusions. From the Bayesian perspective, this uncertainty may be overcome by using Sequential Bayes Factor Designs (Schönbrodt et al. 2017)⁠. Rather than specifying an (unrealistic) alternative hypothesis, researchers may sample sequentially more and more subjects (or trials in a single-subject design) until a pre-defined level of evidence for either the null or alternative or both has been reached. To our knowledge, these Bayesian sampling plans have not been used for *1D*- data and will require further investigation. For the presented data, a slight disadvantage of the Bayesian SPM is computation time which is shown in Table 4. The frequentist calculation times are practically zero (analytic solutions exist), while a negligible but non-zero computation time is required for the Bayesian results. The computation time increases for more complex designs because they require the calculation of several Bayes Factor objects corresponding to several potential alternative hypotheses (in the *t*-test case, only one alternative hypothesis was used). The size of the datasets did not seem to impact the calculations, so for most biomechanical applications (typical sample sizes <100), the computational burden is expected to be no real problem.

**Table 4.** Computation time for frequentist and Bayesian SPM tests

Statistical test and dataset size	Computing time *(DELL, i7 processor, Linux Mint 19 operating system)*
Independent-sample *t-*test SimulatedTwoLocalMax [12 x 101]	Classical SPM (Python): 0.025 s Bayesian SPM (RStudio): 1.325 s
Paired-sample *t-*test PlantarArchAngle [20 x 101]	Classical SPM (Python): 0.025 s Bayesian SPM (RStudio): 1.097 s
Paired-sample *t*-test GaitSymmetry [198 x 101]	Classical SPM (Python): 0.025 s Bayesian SPM (RStudio): 1.580 s

Much further work needs to be performed to explore the validity and theoretical properties of this Bayesian SPM and the FDR control schemes of thresholding on the posterior probability and *q*-values. Bayesian inference and decision-making are not based on controlling type I or type II error rates, and the problem of multiple testing is, therefore, less a problem than in frequentist inference (Berry and Hochberg 1999; Kruschke and Liddell 2018b). In *0D* statistics, *p*-values are typically less conservative than Bayesian methods. In datasets one and two, we saw that the clusters based on the conservative threshold were indeed smaller than the frequentist clusters, but the q*-based clusters were a little larger than the classical ones. For the third dataset, the comparison between cluster sizes is difficult because they really signify different conclusions. Friston and Penny (2003) compared Bayesian 95%-thresholded posterior probability maps with SPM (PET and fMRI data) and saw that the Bayesian approach yielded larger supra-threshold clusters than classical SPMs.

In the future work, we should examine the feasibility of hierarchical Bayesian modeling (empirical parametric Bayes) for *1D* data (Friston and Penny 2003)⁠ which can be used to practically eliminate the problem of multiple testing (Gelman et al. 2012)⁠. Future studies should also examine more appropriate priors for spatiotemporally correlated data for SPM applications (Lee et al. 2017; Sidén et al. 2017)⁠. In the present proposition, we took a default JZS prior at each point in the time series which does not take the temporal correlation into account. One possibility we see in this respect is the introduction of a ‘dynamic prior’ where the posterior density at t_i_ can serve as the prior for the Bayes Factor calculation at t_i+1_. Given the temporal correlation of the data, the effect sizes at neighboring time samples are bound to be similar and therefore the posterior at t_i_ will be a good estimate for that at t_i+1_.

## Supplementary Material

Supplemental Material
